# To eliminate police or redirect police funds? The impact of framing on public support for defunding the police

**DOI:** 10.3389/fpsyg.2024.1439463

**Published:** 2024-09-02

**Authors:** Olivia K. H. Smith, Cassandra Flick, Nicholas D. Michalski

**Affiliations:** ^1^Department of Psychology, University of South Carolina Aiken, Aiken, SC, United States; ^2^Department of Psychology, University of North Dakota, Grand Forks, ND, United States; ^3^Department of Psychology, University of Wyoming, Laramie, WY, United States

**Keywords:** defund the police, framing, public, support, eliminate, redirect

## Abstract

**Objectives:**

Previous research and public polls demonstrate the public has varying levels of support for Defund the Police (DTP). However, much of these results assess how individuals *feel* about DTP rather than how they *define* it. Additionally, the literature fails to consider the role of these definitions and framing in individuals’ assessments of DTP. Using both qualitative and quantitative methodology, the current studies assess the following research questions: (1) How do individuals define DTP and to what extent do individuals support it? and (2) Does framing DTP as either redirecting police funds or eliminating police impact level of support?

**Method:**

Participants in both Study 1 (*N* = 93) and Study 2 (N = 494) were recruited from Amazon Mechanical Turk. Study 1 used a nonexperimental design, in which participants provided their definition of DTP and reported their level of support for DTP. Study 2 used a two-group experimental design in which participants were randomly assigned to one of two frames for DTP garnered from Study 1: redirect funds or eliminate police. Subsequently, participants reported their DTP support, DTP definition, familiarity with DTP, political orientation, and alignment with the frame presented.

**Results:**

In the absence of a frame (Study 1), more participants opposed DTP than supported DTP. Participants also reported several different definitions of DTP, including decreasing police funds, redirecting police funds to social services, and eliminating police altogether. Notably, how an individual defined DTP was associated with level of support (e.g., defining DTP as redirecting funds was associated with greater support for DTP, compared to this theme being absent in participant definitions). When using the redirect theme and eliminate theme as experimental frames (Study 2), a causal relationship was found between the framing manipulation and support of DTP (i.e., the redirect frame led to greater support than the eliminate theme).

**Conclusion:**

The findings from the current studies shed light on how individuals conceptualize DTP, and most importantly, they provide evidence that differential framing can impact support for DTP in the general population. These results have implications for police reform advocates in that the words used to describe DTP can have an impact on public buy-in of policies.

## 1 Introduction

Following the killing of George Floyd by a Minneapolis police officer in 2020, protests erupted across the nation in response to the excessive use of force and apparent racial discrimination (Smith, 2020). In addition to calls for criminal prosecution of the officers involved in Floyd’s death, the public also demanded immediate police reform. Yet, activists argued that police reform as previously conceptualized (e.g., implicit bias training, emphasis on individual accountability, etc.) was insufficient (Kaba, 2020). For instance, the officer who killed George Floyd had over 17 misconduct complaints on his record (Kaba, 2020), providing evidence of another police officer working in a system with a lack of accountability. Given the history of unsuccessful police reforms and calls by activists for changes in policing beyond simply reform (Black Lives Matter Global Network, 2020), individuals and activists popularized the now well-known slogan of “defund the police” (DTP). Millions fled to the streets in response to Floyd’s death and the deaths of other unarmed Black civilians (e.g., Breonna Taylor in Louisville, KY) to protest police violence and advocate for calls to DTP. Given the surge in public interest with the DTP movement, public polling companies (e.g., Pew Research Center) began measuring public sentiment toward different police reforms, particularly regarding the use and amount of police funds. In 2020, 25% of Americans supported a decrease in funding for the police, with 42% stating that funding should stay the same, and 31% supporting an increase in police funds (Pew Research Center, 2020). Empirical research using survey methodologies suggests similarly split results (e.g., Jackson et al., 2023). For example, 43.3% of a nationally representative 677-person sample self-reported that they supported defunding the police and reinvesting in services for the community (Baranauskas, 2022). Notably, division on the issue appears split between political party lines and age groups, with evidence emerging from both public opinion polls and empirical survey research. Generally, both Republican and older individuals independently indicate greater opposition for police reform and DTP (e.g., Baranauskas, 2022; Rakich, 2020). For instance, data from 2020 suggests that individuals over 50 years old are less likely to support decreases in police funding than individuals under 50 years old (Pew Research Center, 2020).

While examining results from polls and survey research can provide information on public sentiment, it cannot answer what the public believes the slogan DTP truly means. For instance, when answering polling questions about DTP attitudes, what do American citizens believe the term implies? In other words, what is DTP actually advocating for? From an official stance of the Black Lives Matter (BLM) leaders, DTP is an abolitionist movement that primarily calls to divest funds from police departments and invest those funds into other local community resources with the hope that this investment will result in better outcomes for community members (Black Lives Matter Global Network, 2020; Perano, 2020). From a BLM standpoint, the overarching sentiment of DTP is that previous attempts to reform the police in the United States have failed; thus, the only option is to envision a new society with increased community services, where police are obsolete (Kaba, 2020). Ultimately, the hope is that diverting community funds from the police to other social services, such as education and mental health, will improve the communities and decrease crime (Black Lives Matter Global Network, 2020). Yet, there is a lack of research examining the public’s understanding of the meaning of DTP (for an empirical exception see Cobbina-Dungy et al., 2022 explained below). Further, there is a gap in the research examining how psychological framing of DTP may impact individual’s understanding of the slogan and support. Given the relevance of these questions to today’s policing climate in the United States, we used multiple methodologies to (1) qualitatively examine the public’s definition of DTP, and (2) quantitatively examine how framing influences public support for and against definitions of DTP.

### 1.1 Public perception of defund the police

Although support for the DTP movement increased following nationwide protests in 2020, DTP is not a new concept or call to action. Lethal police force incidents have occurred prior to George Floyd and continue to occur. For instance, in 2014, community protests began in Ferguson, Missouri following the death of Michael Brown (Lopez, 2016), and in 2023, Tyre Nichols was violently beaten to death by Memphis police officers (Cochrane and Rojas, 2024), reminding advocates and the public that previous attempts at police reform have remained largely unsuccessful. Some scholars have suggested that we should view police through the lens of racialized social control, and we should reform our system to reallocate resources to community-based solutions (Cobbina-Dungy et al., 2022). Thus, calls to DTP continue, and much research over the past few years has examined public support and individual characteristics that have the potential to exacerbate or mitigate that support.

Much of the research examining public perception of DTP assesses perceptions by posing quantitative questions concerning support. For instance, Baranauskas (2022) sampled 677 individuals, and asked them how much they agreed with the following statement: “The best way to deal with crime in general is by defunding the police and re-investing in social services.” While this statement certainly measures individuals’ support for re-investing funds in social services, it fails to capture individuals’ personal perception of what DTP means. Nevertheless, these results provide valuable information regarding public sentiment about DTP. Data indicated that support for DTP (i.e., re-investment into social services in this case), was mixed, and conservatives were less likely to support this proposition, while minorities were more likely to express support (Baranauskas, 2022). Additional research on the matter of political partisanship suggests that the differential support by party identity is rooted in the interpretation of DTP (Jackson et al., 2023). Liberal individuals are more likely to perceive DTP to mean reallocation, while conservative individuals are more likely to perceive DTP to mean eliminate police altogether (Jackson et al., 2023). Additional research indicates that the primary concern that individuals have when considering the ramifications of DTP is crime and safety (Vaughn et al., 2022). Interestingly, individuals tend to be less in favor of proposals that would reduce police response to calls as individuals consider this a threat to public safety.

Outside of public perceptions and polling studies, scholars have begun to examine sub-sets of the population to obtain a more thorough understanding of the support landscape. For instance, Fine and Toro (2022) surveyed a national sample of adolescents to determine how they viewed defunding and abolishing the police, separately. Results indicated adolescents were more in favor of defunding the police, rather than abolishing, suggesting that phrasing is important when considering police reform. Additionally, the data showed that support for abolishing the police increased when adolescents received “the talk” (i.e., how to behave when approached by police) more often.

In contrast to the previous studies, Cobbina-Dungy et al. (2022) took a qualitative approach to examine public perception of DTP by conducting qualitative interviews with 28 individuals who attended the Commitment March in 2020, a march held in Washington D.C. protesting excessive force against Black individuals. In addition to questions about police interactions, individuals responded to questions asking them to define DTP. The majority of the sample defined DTP as reallocating resources from police departments to community resources, rather than abolishing the police (Cobbina-Dungy et al., 2022). Importantly, some of the sample expressed concern about the slogan “defund the police,” suggesting it may act as a distraction and barrier to reform due to the negative connotation. For example, one protestor stated, “People get so scared when they hear defund the police, because they are like, ‘Oh, well, everyone’s going to wreak havoc and there’s not going to be law and order anymore,’ and that’s not the case” (Cobbina-Dungy et al., 2022, p. 159). Given this sentiment combined with previous research demonstrating that support for policies is largely rooted in framing (Nelson et al., 1997), it is important to consider what DTP signals to the public. In other words, to assess support for DTP, we must also understand how framing may or may not impact individuals’ perceptions.

### 1.2 Framing effects

While DTP-related experts, such as BLM leaders, discuss the slogan’s meaning as a abolitionist strategy of redirection of community funds from police departments to other social services (Black Lives Matter Global Network, 2020), it is still largely unclear how the public perceives the slogan. This is particularly important because the way a social or political issue is defined or framed can have an impact on public support for policies related to that issue (e.g., Bruckmüller et al., 2017; Chow and Galak, 2012; Clarke et al., 2015). Frames can be conceptualized as a way to “select some aspects of a perceived reality and make them more salient in communicating text” (Entman, 1993, p. 52), and they can serve as a “bridge between elite discourse about a problem or issue and popular comprehension of that issue” (Nelson et al., 1997, p. 224). Thus, individuals in positions of power (e.g., the media) may selectively choose which pieces of information about a policy issue to include in their coverage, thus framing the issue in a certain light (Iyengar and Simon, 1993; Moy et al., 2016). This is extremely relevant to the DTP as it has been frequently covered by various media outlets since 2020 (e.g., Novacic, 2021).

The impact of framing has largely been examined in the psychological literature since the seminal work of Tversky and Kahneman (1981) that suggested the way in which information is presented (e.g., the formulation of alternatives) can significantly impact decision-making. The original work focused on the framing of risky choices, such as being framed in terms of losses versus gains (e.g., Levin et al., 1998; Tversky and Kahneman, 1981). Subsequent work has focused on the presentation of identical information in two separate ways. For instance, in one experiment researchers asked participants to rate the quality of ground beef. The researchers manipulated whether the fat content in the beef was framed in terms of the percentage of lean content (i.e., 75% lean) or percentage of fat content (i.e., 25% fat). Results indicated that participants rated the quality of the beef as better in the 75% lean frame, compared to the 25% fat frame, even though the fat/lean content was equivalent across conditions, known as *equivalent* framing (Levin and Gaeth, 1988). Given the proliferation of framing research (Entman, 1993), framing effects have since been found across a wide variety of judgments, such medical and financial decisions (e.g., Haward et al., 2008; Kühberger et al., 2002), as well as perceptions of political and social policies (e.g., Bruckmüller et al., 2017; Chow and Galak, 2012; Gifford and Comeau, 2011).

Importantly, framing of social political issues can impact support for policies (Nelson et al., 1997), such as immigration. For instance, some research indicates that when participants are exposed to a frame referencing increased crime as a consequence of immigration (negative contribution frame), compared to a frame emphasizing economic growth (positive contribution frame), they have more negative views of immigration (Igartua and Cheng, 2009). Similar frames have been found to impact public sentiment regarding government spending (Jacoby, 2000), war decisions (Iyengar and Simon, 1993), and capital punishment (Peffley and Hurwtiz, 2007).

The examples described above, and the examination of framing we take in the current work, can be conceptualized as the impact of *emphasis* framing (Druckman, 2001; also referred to as issue framing). With emphasis framing, researchers examine the extent to which “emphasizing a subset of potentially relevant considerations” may impact the way in which an individual forms their opinions about a program or policy (Druckman, 2004, p. 672). The notion of equivalency is not necessary for framing effects to emerge in the context of emphasis framing. In other words, emphasis frames “focus on qualitatively different yet potentially relevant considerations” (Druckman, 2004, p. 672). For example, in a recent experiment, researchers asked participants to rate how much they endorse various police reform policies (e.g., body cameras and civilian oversight boards) following the presentation of 1 of 3 frames: racial injustice (i.e., racial disparities in excessive force incidents are due to police treating African Americans unfairly), differential involvement (i.e., racial disparities in excessive force incidents are due to African Americans’ disproportional involvement in criminal activities), or a control frame (i.e., excessive force incidents have initiated discussions about police reform). Results indicated a significant impact of framing when considering participant’s pre-existing attitudes toward the issues presented in the frames (Dunbar and Hanink, 2023). Specifically, participants who held attitudes similar to the framing manipulation they were exposed to were more (the racial injustice frame) or less (the differential involvement frame) likely to support police reform efforts (Dunbar and Hanink, 2023).

Thus, framing of information and issues by emphasizing different aspects of an issue can have significant consequences for individuals’ perceptions of the information. These effects have been found across various types of decisions (e.g., medical decisions, risk decisions), as well as more general perceptions and evaluations. It seems possible that the issue of DTP may also be susceptible to framing effects, such that emphasizing particular considerations about DTP may impact evaluation (i.e., support), or even conceptualizations, of the movement. Initial research suggests this may be the case. For example, Paulson-Smith et al. (2023) found that participants are less supportive of DTP when the slogan “defund the police” is used compared to “Using some of a police department’s budget to fund community policing and social services” (Paulson-Smith et al., 2023, p. 6). Although this study initiates an important conversation about wording and frames surrounding DTP, it fails to inform us of how individuals define DTP and how these definitions alongside the DTP slogan could impact support. Given the lack of examination of how the public defines DTP, it remains unclear as to what information or considerations may be most susceptible to framing effects. In the current research, we explore these unanswered questions.

### 1.3 Current research

While the BLM movement largely defines DTP as the reallocation of funds from police departments to community resources as an abolitionist strategy, it is still uncertain how the public defines DTP. Public polling and survey research has demonstrated mixed support for DTP, but this methodology lacks the qualitative component that could provide a clearer understanding of DTP. While Cobbina-Dungy et al. (2022) explored this qualitatively, they did so with a sample of individuals that had just attended a public protest. Although it is important to understand this subset of the population, it is possible that these individuals’ judgments may differ from the general U.S. public. Given this, we sought to collect a broader sample in the current study to better understand individuals’ conceptualizations of DTP through qualitative and quantitative methods (Study 1). We then used these conceptualizations to experimentally manipulate emphasis framing of the slogan and measure the impact on participants’ support and definitions of DTP (Study 2).

## 2 Study 1

Prior to examining the impact of framing on individuals’ support of DTP, Study 1 sought to determine what relevant considerations related to DTP exist by measuring lay definitions of DTP and exploring if certain themes in definitions may be associated with self-reported support for the movement. Thus, we explored the following questions: (1) How do individuals define DTP in their own words? (2) To what extent do individuals support DTP? and (3) Is the way in which individuals define DTP associated with their level of support for DTP? For instance, while previous research indicates that liberal individuals are more likely to conceptualize DTP as reallocating some police funds to other social services (e.g., Jackson et al., 2023), and other research suggests conservatives are typically less supportive of DTP (e.g., Baranauskas, 2022), it remains empirically unexamined whether varying definitions may be the reason for differential support. In Study 1, we sought to begin exploring this question by examining whether and to what extent variations in individuals’ definitions of DTP were associated with varying levels of support, within the same sample of participants.

### 2.1 Method

#### 2.1.1 Participants

Participants were recruited via Amazon’s Mechanical Turk (*Mturk*) in May 2021. An open-ended attention check question asking participants what they did during the study was used to assess the quality of their responses. After removing those that failed the attention check (i.e., those who failed to write something relevant to what they did in the study, *n* = 6), our final sample included 93 participants, 55.9% female and 44.1% male, with ages ranging from 21 to 73 years old (*M* = 43.67, SD = 13.32). Participants identified as 73.1% Caucasian/White, 9.7% Asian/Pacific Islander, 8.6% Black/African American, 7.5% Hispanic/Latino, and 1.1% other/multi-racial, and overall, the sample leaned slightly liberal (*M* = 3.63, SD = 1.93, on a 7-point scale 1 = *extremely liberal*, 7 = *extremely conservative*). Participation took approximately 15 min, and participants were compensated $1.00 USD.

#### 2.1.2 Measures

##### 2.1.2.1 Definition of defund the police

Participants’ definitions of DTP were assessed via an open-ended question that stated, “How would you define ‘defund the police’”?

##### 2.1.2.2 Support for defund the police

Similar to Jackson et al. (2023), participants reported their level of support or opposition for DTP on a 5-point Likert scale ranging from 1 (*strongly oppose it*) to 5 (*strongly support it*).

#### 2.1.3 Procedure

Participants were instructed that they would answer questions regarding their perceptions of DTP. Following informed consent, participants completed the measures described above. Participants then completed demographic questions and the open-ended attention check before receiving their completion code for payment.

### 2.2 Results

#### 2.2.1 Definitions of defund the police

Our final sample consisted of 93 codable participant responses which were coded by the three authors. Relevant coding themes were determined for the open-ended question following an initial review of participant responses and discussion among the authors. The following four themes emerged as definitions for DTP: decrease, redirect, eliminate police, and emotion. Each theme was coded as present (1) or absent (0). Response coding was not mutually exclusive, in that participant responses could be coded for the presence of multiple themes at once. Fleiss’ kappa, a statistic used for measuring the reliability of coding agreement between three or more raters, was utilized. Fleiss’ kappa measures the difference between observed and expected agreement among coders (Hoang et al., 2018). Kappa values are categorized into the following discrete categories: < 0 indicates less than chance agreement, 0.1–0.2 indicates slight agreement, 0.21–0.40 indicates fair agreement, 0.41–0.60 indicates moderate agreement, 0.61–0.80 indicates substantial agreement, and 0.81–0.99 indicates almost certain agreement (Fleiss, 1971; McHugh, 2012). In line with these classifications, kappa agreement between the raters in our study ranged from moderate to almost certain (redirect = 0.96, decrease = 0.90, eliminate = 0.83, emotion = 0.60). Raw agreement between raters ranged from 83.9 to 98.9%. Discrepancies between raters were discussed by the research team and resolved through consensus.

##### 2.2.1.1 Decrease

The “decrease” theme (87.1%) was coded as present when a response included the idea of decreasing money given to the police or police departments. Examples of responses coded for the presence of decrease include: “to take away funds from the police officers” (participant 46), “taking funds away from the police” (participant 54), and “take money and resources away from the police so their power and authority is reduced” (participant 61). Among participants that mentioned the decrease theme in their definition, 58.5% also mentioned redirect, 21.9% mentioned eliminate, 6.1% mentioned emotion, and 20.73% did not mention any other theme.

##### 2.2.1.2 Redirect

The “redirect” theme (51.6%) was coded as present when a response mentioned not only decreasing funding toward the police but also redirecting or diverting those funds to other community services (e.g., social services, mental health resources, etc.). Thus, if a response was coded as “redirect” it must also have been coded for “decrease”; however, the opposite is not true. Examples of responses coded for the presence of redirect include: “diverting some money from the police force to other areas, such as social services” (participant 4) and “taking money used for police officers and using that money for other resources such as social workers and mental health professionals to help with crime” (participant 66). Among those who mentioned the redirect theme in their definition, 6.3% also mentioned eliminate and 2.1% mentioned emotion.

##### 2.2.1.3 Eliminate police

The “eliminate police” theme (22.6%) was coded as present when a response mentioned the idea of completely removing, or eliminating, all police forces and/or all funds for policing. Examples of responses coded for the presence of eliminate include: “remove the police and take away their pay” (participant 33), “remove the funds to police and self-govern” (participant 51), and “replace all the police with social workers” (participant 9). For those participants who mentioned the eliminate theme, 14.3% mentioned the redirect theme, 85.7% mentioned decrease, 19.1% mentioned emotion, and 9.5% mentioned no other theme.

##### 2.2.1.4 Emotion

The “emotion” theme (14%) was coded as present when a response included the presence of strong emotion or a strong opinion toward the subject of DTP. These responses sometimes included a true response to the questions posed, for example, “stop giving them money to do jobs they suck at that end with the deaths of innocents” (participant 43), and sometimes did not, “a stupid idea that all the liberals are just eating up” (participant 32). Among those that included the emotion theme in their definitions, 7.7% mentioned redirect, 38.5% mentioned decrease, 30.8% mentioned eliminate, and 53.9% mentioned no other theme.

#### 2.2.2 Support for defund the police

On average, participants had greater opposition, rather than support, for defunding the police (*M* = 2.35, SD = 1.56, on a 5-point scale), with 61.3% self-reporting they opposed defund the police (48.4% strongly opposed, 12.9% opposed) and 30.2% reporting they supported defunding the police (15.1% strongly support, 15.1% support).

#### 2.2.3 Do definitions of defund the police predict level of support?

To assess the extent to which participants’ DTP definitions were associated with differential levels of support, we conducted a series of t-tests examining whether presence of certain themes in their qualitative definitions were associated with their self-reported levels of support. To account for unequal variances and multiple comparisons, the *t* statistics reported below rely on un-pooled variances and a Bonferroni correction (i.e., *p*-values compared to a cutoff <0.0125).

##### 2.2.3.1 Redirect theme

The presence of the “redirect” theme in participants’ definition was associated with their level of support, *t* (88.36) = 4.40, *p* < 0.001, *d* = 0.91. Those who defined defund the police as redirecting funds (*n* = 48) indicated greater levels of support for the movement (*M* = 2.98, *MOE* = ± 0.46, *SD* = 1.58) compared to those whose definitions did not include the theme of redirect (*n* = 45; *M* = 1.69, *MOE* = ± 0.37, *SD* = 1.24). The mean difference was 1.29 with a 95% CI ranging from 0.70 to 1.88.

##### 2.2.3.2 Eliminate theme

The presence of the “eliminate” theme in participants’ definition was associated with their level of support, *t* (47.94) = −3.78, *p* < 0.001, *d* = −0.76, but in the opposite direction of the “redirect” theme. Those who defined defund the police as eliminating police (*n* = 21) indicated less support for the movement (*M* = 1.48, *MOE* = ± 0.66, *SD* = 1.08) than those whose definitions did not include the eliminate theme (*n* = 72; *M* = 2.61, *MOE* = ± 0.37, *SD* = 1.59). The mean difference was −1.14 with a 95% CI ranging from −1.74 to −0.53.

##### 2.2.3.3 Decrease theme

The presence of the “decrease” theme in participants’ definition was associated with their level of support, *t* (35.48) = 4.54, *p* < 0.001, *d* = 0.77. Participants who included the theme of “decrease funds” in their definitions (*n* = 81) indicated greater levels of support for defund the police (*M* = 2.51, *MOE* = ± 0.36, *SD* = 1.60) compared to those whose definitions did not include the decrease theme (*n* = 12; *M* = 1.33, *MOE* = ± 0.41, *SD* = 0.65). The mean difference was 1.17 with a 95% CI ranging from 0.65 to 1.70.

##### 2.2.3.4 Emotion theme

The presence of the “emotion” theme in participants’ definition was not significantly associated with their level of support, *t* (17.93) = −2.11, *p* = 0.049.

### 2.3 Study 1 discussion

Study 1 provided preliminary evidence about how laypersons conceptualize the DTP movement. There was considerable discrepancy in how individuals defined DTP. A majority of participants (87.1%; decrease theme) defined the slogan as decreasing some amount of money from the current police forces. Although this aligns with Cobbina-Dungy et al. (2022) results, participants showed various nuances in their definitions as well. Specifically, there was considerable inconsistency among participants about whether DTP meant taking *some* money from police and then redirecting those funds to other community services (51.6%; redirect theme), or whether it meant taking *all* funding from police and eliminating the police force altogether (22.6%; eliminate theme). Given this discrepancy in defining what the slogan means, the variation in support for the movement, and one of our questions of interest, we explored the extent to which how participants defined DTP might be related to their level of support for the movement. Results suggested that defining DTP as reducing funds given to the police department and redirecting those funds to other social services was associated with greater levels of support for DTP. Conversely, defining the term as eliminating all funding for police and getting rid of the force altogether was associated with decreased support.

Thus, two main conclusions can be garnered from Study 1. First, layperson-generated definitions of DTP lead to the emergence of two overarching themes that varied across definitions – the redirect theme and eliminate theme – that may result in framing effects of DTP. Second, our findings provide some initial empirical support for the notion that variations in individuals’ definitions of DTP might be associated with varying levels of support for the movement. Recall that previous research provides evidence suggestive of this (e.g., Jackson et al., 2023; Baranauskas, 2022), yet this had been largely unexamined in an empirical context. However, given the correlational nature of Study 1, it is also possible that individuals with lower levels of support for DTP are potentially less motivated to provide compelling or descriptive definitions of DTP. In other words, it is unclear if different definitions for DTP are driving variations in support level (as suggested above) or vice versa. Thus, we address this concern in Study 2.

## 3 Study 2

To further examine the association between individuals’ definitions and support found in Study 1, Study 2 explored the extent to which exposure to public-generated themes associated with DTP may result in differential levels of support for the movement, supplementing the correlational nature of Study 1 with a potential causal relationship. Specifically, two themes discovered in Study 1 were chosen as experimental manipulations: Redirect and Eliminate themes. These themes were selected due to their relevance to previous public polling and Study 1 results. Specifically, the emotion theme was uncommon among participants in Study 1, and although the decrease theme was common, this was likely influenced by the redirect category. In other words, because the qualitative coding was not mutually exclusive, individual responses coded as redirect were always also coded as decrease (due to the decrease in funds being inherent to redirection of funds). Thus, the percentage of definitions with the decrease theme present in Study 1 is inflated. Given this, and the variation in participant definitions regarding the redirect and eliminate themes, we elected to utilize these as our frames for Study 2. Based on Study 1 results and related findings from Paulson-Smith et al. (2023), we predicted participants presented with the redirect framing condition would express greater levels of support for DTP than participants exposed to the eliminate framing condition (Hypothesis 1). Similar to Study 1, we were also interested in the extent to which exposure to the redirect or eliminate condition might impact participants’ own definitions of DTP. Based on Study 1 results, we predicted that participants presented with the redirect frame would be more likely to include the redirect theme in their own definitions of DTP (Hypothesis 2a), while participants presented with the eliminate theme would be more likely to include the eliminate theme in their own definitions of DTP (Hypothesis 2b).

Lastly, DTP is a popularized slogan that has garnered a great deal of public attention since 2020. Given our primary dependent measure – support for DTP – and our main question of interest examining how these frames may impact that support, we felt it necessary to control for a handful of other relevant factors and individual differences that may be associated with individuals’ support for DTP in our study. Specifically, we measured and controlled for participants’ political orientation, given that variations in political orientation are associated with varying levels of support for DTP. Based on previous research (e.g., Baranauskas, 2022; Jackson et al., 2023; Rakich, 2020), we predicted that more conservative participants would report lower levels of support for DTP compared to less conservative participants (Hypothesis 3a). Based on additional previous research examining political orientation and perceptions of DTP (e.g., Jackson et al., 2023), we also predicted more conservative participants would be more likely to include the eliminate theme in their definitions compared to less conservative participants (Hypothesis 3b). Finally, we measured and controlled for participants familiarity with DTP and the extent to which our experimental framing definition aligned with their pre-existing definition of DTP when entering the study. These measures were included primarily as control variables. In other words, measuring these concepts and controlling for them in our analyses allowed us to examine the impact of our experimental manipulation on levels of support, *regardless of* how familiar participants were with DTP coming into the study and how much their own previous conceptualization of DTP aligned with the definition we provided them in our study. Thus, the impact of these control variables (familiarity and alignment) on level of support for DTP were exploratory, and therefore we did not have specific hypotheses regarding their potential impact.

### 3.1 Method

#### 3.1.1 Participants and design

An *a priori* power analysis using WebPower package (Zhang and Yuan, 2018) in R 4.1.2 (R Core Team, 2022) indicated that a sample size of 198 would allow for sufficient power (0.80) to detect a medium effect (*f*^2^ = 0.25, Cohen, 1988). Although Study 1 results indicated a large effect of theme presence (e.g., redirect funds or eliminate police) on support, we took a conservative approach by using a medium effect for our power analysis. We oversampled and recruited 500 participants via *Mturk* in May 2022 anticipating that we would have to remove some participants for inattention. After removing those that failed our open-ended attention check (*n* = 6), the final sample was 494 participants, 53.2% male, 46.2% female, and 0.6% who identified as other. Participant ages ranged from 20 to 84 years old (*M* = 41.74, SD = 11.91). Our sample was s 72.7% Caucasian/White, 11.9% Black/African American, 5.7% Asian/Pacific Islander, 5.5% Hispanic/Latino, 1.0% Native American/Alaska Native, and 3.2% other/multi-racial. Our sample leaned slightly liberal in terms of self-reported political orientation (*M* = 3.63, SD = 1.75, on a 7-point scale 1 = *extremely liberal*, 7 = *extremely conservative*). Aside from political orientation and a lower percentage of participants identifying as Hispanic/Latino, the sample mirrors the United States demographic landscape as reported by the U.S. Census Bureau (U.S. Census Bureau, 2024). Participation took approximately 10 min and participants were compensated $1.00 USD.

We used a two group between-subjects experimental design in which participants were randomly assigned to one of two different frames of defund the police: *redirecting funds* or *eliminating police*.

#### 3.1.2 Procedure, materials and measures

Participants were instructed that they would answer questions about their perceptions of policing. Following informed consent, participants’ familiarity with DTP was assessed prior to being randomly assigned to one of the two framing manipulations described below (eliminate police or redirect police funds). Following exposure to the description of DTP, participants then indicated their support for DTP, provided their own definition of DTP, and indicated the extent to which the provided definition aligned with their personal definition. Participants then completed demographic questions and the open-ended attention check, were thanked and given their completion code for payment.

##### 3.1.2.1 Framing manipulation

Participants were randomly assigned to receive one of two different definitions of defund the police, which framed the movement as either eliminating police or redirecting police funds to social programs. Framing manipulations included a brief written summary accompanied by an audio recording (~ 1.5 min). In the eliminate police condition, “defund the police” was defined as eliminating or completely removing traditional police forces in communities. In the redirect condition, “defund the police” was defined as reallocating or redirecting funds away from police departments to other government agencies and social programs, to address social issues such as mental health, addiction, and homelessness.

##### 3.1.2.2 Support for defund the police

Participants reported their level of support or opposition. For DTP on the same 5-point Likert scale from Study 1.

##### 3.1.2.3 Definition of defund the police

Similar to Study 1, participants were asked to provide their own personal definition of “defund the police” (i.e., not simply reiterating the definition they were presented). To do this, we utilized the following open-ended question similar to the first two studies: “*In your opinion, what does “defund the police” mean? In other words, how do you define defund the police?*”

##### 3.1.2.4 Control measures

We included measures assessing participants familiarity with the DTP movement, as well the extent to which the definition that we provided them with aligned with their pre-existing personal definition. To measure familiarity, we asked participants how much they have heard or read about DTP on a 4-point Likert scale ranging from 1 (*not at all*) to 4 (*a lot*) (Clarke et al., 2015). To assess similarity between our provided definition and participant’s personal definition, participants were asked to rate the extent to which their own personal definition of “defund the police” aligned with the definition they were presented with on a 5-point Likert scale ranging from 1 (*not at all*) to 5 (*greatly aligns*).

##### 3.1.2.5 Manipulation check

To ensure participants were aware of the definition they received, they were asked the following multiple-choice question, “the information presented to you defined “defund the police” as: eliminating police, increasing police training, reallocating funds to social services, or increasing military equipment for police. Analyses indicated our manipulation was salient in that 95.55% (*n* = 472) of our sample provided the correct response.

### 3.2 Results

#### 3.2.1 Descriptive statistics

On average, participants had slightly greater opposition, compared to support, for DTP (*M* = 2.52, *MOE* = ± 0.14, *SD* = 1.49, on a 5-point scale), with 55.7% self-reporting they opposed defund the police (38.1% strongly opposed, 17.6% opposed) and 30.6% reporting they supported defunding the police (14.8% strongly support, 15.8% support). Participants were quite familiar with the defund the police slogan (*M* = 3.21, *MOE* = ± 0.06, *SD* = 0.74, on a 4-point scale), with 38.5% indicating they have heard “a lot” about DTP, and only 1.8% indicated they had not heard about DTP. Participants indicated that their experimental framing condition somewhat aligned with their pre-existing definition of DTP (*M* = 3.46, *MOE* = ± 0.14, *SD* = 1.43, on a 5-point scale), with 28.9% indicating it greatly aligned and 17.6% stating it did not align at all. For descriptive statistics of participants’ support for defund the police, familiarity with the movement, and alignment by each experimental framing condition, see Table 1.

**Table 1:** Means and standard deviations for study 2—dependent measures by experimental condition.

	Redirect frame		Eliminate frame		
Measure	*M*	SD	*M*	SD	Range
Support for DTP	2.90	1.49	2.14	1.39	1–5
Familiarity with DTP	3.22	0.71	3.20	0.78	1–4
Alignment of definitions	3.87	1.21	3.05	1.52	1–5

*N for redirect frame = 245; N for eliminate frame = 249.*

For definition coding, we coded participants’ responses to the question, “How would you define ‘defund the police’”? using a coding scheme that differed slightly from Study 1. The themes coded for in Study 2 were: codable, redirect, decrease, and eliminate. The emotion theme was removed given the low frequency of responses containing the theme in Study 1 and the lack of significant effect on support. Codes of redirect, decrease, and eliminate were coded in the same way as the previous study. Codable denoted whether participants provided a legitimate attempt to answer the posed question, or a response that did not align with the question prompt (e.g., “Make sure our beliefs stay strong and getting help for those who need it,” participant 319). Similar to Study 1, results indicated “decrease” was the most prevalent theme (69.03%) in participants’ definitions, followed by the “redirect” theme (50.80%), and “eliminate” theme (27.13%).

#### 3.2.2 Does framing impact support for DTP?

A multiple linear regression model examining the effect of framing manipulation on support for DTP, while controlling for participant’s familiarity with DTP, political orientation, and alignment of definitions, was significant, *F* (4, 489) = 63.29, *p* < 0.001, *R*^2^ = 0.34. There were significant main effects of framing (*b* = 0.59, *SE* = 0.11, *p* < 0.001, η_p_^2^ = 0.05), political orientation (*b* = −0.42, *SE* = 0.03, *p* < 0.001, η_p_^2^ = 0.27), and alignment (*b* = 0.17, *SE* = 0.04, *p* < 0.001, η_p_^2^ = 0.03). Participants in the redirect condition indicated greater support for DTP by an average increase of 0.59 points on a 5-point scale, compared to those in the eliminate framing condition. For every one-unit increase in conservatism, participants support for DTP decreased by an average of 0.42 points. For every one-unit increase in alignment of definitions, participants’ support for DTP increased by an average of 0.17 points. Participants’ level of familiarity with DTP did not significantly predict support (*p* = 0.43).

#### 3.2.3 Does framing impact definitions of DTP?

We conducted a series of logistic regressions to examine whether our framing manipulation significantly predicted the presence or absence of our coding themes in participants’ definitions. Similar to the analyses on support for DTP, we controlled for participants’ familiarity with DTP, political orientation, and how much the definition we provided them with aligned with their pre-existing definition of DTP. To account for multiple tests given our three coding themes, a Bonferroni correction was used (i.e., *p*-values compared to a cutoff ≤0.0167). See Table 2 for full model statistics.

**Table 2:** Logistic regression models predicting presence of themes in participants’ definitions of DTP.

	Dependent measure		
	Redirect theme	Eliminate theme	Decrease theme
Condition	0.485^***^	−0.446^∗∗∗^	0.492^∗∗∗^
	(0.039)	(0.035)	(0.036)
Alignment	0.007	0.090^∗∗∗^	−0.045^∗∗∗^
	(0.014)	(0.012)	(0.013)
Familiarity	0.025	0.006	0.066^∗∗∗^
	(0.025)	(0.023)	(0.023)
Political	−0.077^***^	0.055^∗∗∗^	−0.053^∗∗∗^
	(0.011)	(0.010)	(0.010)
(Intercept)	0.444^***^	−0.041	0.585^∗∗∗^
	(0.102)	(0.092)	(0.095)
Observations	494	494	494
Log Likelihood	−263.419	−213.565	−227.586
Akaike Inf. Crit.	536.837	437.130	465.172

^*^p < 0.1; ^**^p < 0.05; ^***^p < 0.01.

##### 3.2.3.1 Redirect theme

The logistic regression on the redirect theme (AIC = 536.84) revealed significant main effects of framing condition and participant political orientation. The odds of the redirect theme being present in participants’ definitions were 63% higher for those in the redirect framing condition, compared to those in the eliminate framing condition. For every one-unit increase in conservatism, the odds of the redirect theme being present in participants’ definitions decreased by 0.08%.

##### 3.2.3.2 Eliminate theme

The logistic regression model on the eliminate theme (AIC = 437.13) revealed significant main effects of framing condition, participant political orientation, and alignment of definitions. The odds of the eliminate theme being present in participants’ definitions were 36% lower for those in the redirect framing condition, compared to those in the eliminate framing condition. The odds of the eliminate theme being present in participants’ definitions increased by 6% for every one-unit increased in conservatism and 7% for every one-unit increase in alignment of definitions.

##### 3.2.3.3 Decrease theme

The logistic regression model on the decrease theme (AIC = 465.17) revealed significant main effects of all predictors in the model: framing condition, political orientation, alignment of definitions, and familiarity with DTP. The odds of the decrease theme being present in participants’ definitions were 63% higher for those in the redirect framing condition, compared to those in the eliminate framing condition. The odds of the decrease theme being present in participants’ definitions decreased by 5% for each one-unit increase in conservatism and for each one-unit increase in alignment (independently). The odds of the decrease theme being present increased by 7% for each one-unit increase in familiarity.

#### 3.2.4 Exploratory model on support

A multiple linear regression model examining the effect of framing condition, political orientation, and the interaction between framing condition on support, while controlling for participant’s familiarity with DTP and alignment of definitions, was significant, *F* (5, 488) = 51.787, *p* < 0.001, *R*^2^ = 0.35. There were significant main effects of framing condition (*b* = 1.08, SE = 0.27, *p* < 0.001, η_p_^2^ = 0.05), political orientation (*b* = −0.36, *SE* = 0.04, *p* < 0.001, η_p_^2^ = 0.28), and alignment (*b* = 0.14, SE = 0.04, *p* < 0.001, η_p_^2^ = 0.02). There was also a significant interaction between framing condition and political orientation (*b* = −0.13, SE = 0.06, *p* = 0.041, η_p_^2^ = 0.01). Participants with political orientation scores at the mean [3.63; *t* (490) = −6.54, *p* < 0.001] or 1.5 *SD*s below the mean [1.05; *t* (490) = −6.15, *p* < 0.001] had greater support for DTP when exposed to the redirect frame compared to eliminate frame. Participants with political orientation scores 1.5 SDs above the mean [6.25; *t* (490) = −1.09, *p* < 0.001] were not significantly impacted by framing condition.

### 3.3 Study 2 discussion

Participants in Study 2 were exposed to one of two framing conditions for DTP: redirect or eliminate. Results indicated that framing had a significant impact on participants’ reported support for the DTP movement, supporting Hypothesis 1. This impact on support was observed after only a brief (~1.5 min) exposure to an emphasis frame of DTP, suggesting that even short descriptions, potentially similar to those most likely to be seen in the United States media, are significantly impacting public support for a social policy. Given that the success of proposed policies can be largely driven by public support (Burstein, 2003), this is noteworthy. It is possible that the effects observed could be larger given prolonged exposure to the frames (Lecheler et al., 2015). Additionally, previous research (Paulson-Smith et al., 2023), has found similar results in that participants are more supportive of DTP when redirecting funds to social services is explicitly mentioned, but the current study adds to this developing body of literature by presenting the phrase “defund the police” along with the frames in both conditions. In other words, even if defund the police has a negative connation (Cobbina-Dungy et al., 2022), the current study suggests following the DTP slogan with an explanation that either frames it as redirecting police funds to social services or eliminating police can have a substantial impact on how individuals assess and report their support for the movement.

Furthermore, findings suggest that the frame affects support beyond pre-existing beliefs regarding familiarity with defund the police (i.e., how much previous exposure participants had to DTP) and alignment (i.e., how much the DTP definition participants were presented with aligns with their personal definition of DTP). While our framing manipulations significantly predicted support, so did political orientation and alignment. Supporting Hypothesis 3a, we found that participants who identified as more conservative, were less likely to support DTP, which aligns with previous research (Baranauskas, 2022).

Beyond support, framing also impacted how individuals chose to define DTP in their own words, supporting Hypotheses 2a and 2b. In other words, the framing manipulation carried over beyond support and subsequently impacted individuals’ definitions of DTP when asked to express the slogan in their own terms. Results indicated participants were more likely to include the presence of the theme associated with the frame that they were exposed to. Similar to the models predicting support, these findings also demonstrated an impact of framing while controlling for important individual differences as well (political orientation, alignment, and familiarity).

In terms of political orientation, participants who were more conservative were more likely to have the eliminate theme present, and less likely to have the redirect frame present, in their definitions. These results support Hypothesis 3b and are in line with previous research (Baranauskas, 2022; Jackson et al., 2023; Pew Research Center, 2020; Rakich, 2020). Interestingly, similar to conservative participants, participants who reported increased alignment were also more likely to have the eliminate theme present in their own definitions. Based on these findings, it appears the effect of alignment on the inclusion of the eliminate theme in participants’ own definitions may be a form of confirmation bias in that participants’ reporting increased alignment with the definition are receiving validation for their belief (Van Swol, 2007). Because eliminate is perhaps the most extreme interpretation of DTP, individuals choosing to define it in that manner may experience confirmation bias, especially considering their beliefs are not supported by the majority (e.g., Study 1 results indicated only 22.6% of the sample defined DTP as eliminating police).

Lastly, while we did not make specific predictions regarding the presence of the decrease theme in participant definitions (due to the high prevalence rate in Study 1 and its relation to the redirect theme), Study 2 findings indicated some interesting results. The presence of the decrease theme was more likely to be present in participant definitions when: (1) participants were presented with the redirect frame, and (2) participants were more familiar with DTP. Given Black Lives Matter defines DTP as divesting funds (i.e., decreasing funds) from police departments and investing those funds into community resources as an abolitionist strategy (Black Lives Matter Global Network, 2020), these results are not surprising. It is logical that participants that were more familiar with DTP would define it, in part, as decreasing funds. In contrast, the presence of the decrease theme was less likely when: (1) participants were more conservative, and (2) participants’ pre-existing definitions aligned more with the frame they were provided with.

In addition to our primary analyses, we conducted an exploratory model to delve further into the effect of political orientation. Since researchers have previously emphasized political orientation as it relates to support for DTP (Baranauskas, 2022; Jackson et al., 2023; Pew Research Center, 2020; Vitro et al., 2022), we thought it was important to explore how the framing manipulation may impact individuals differently based on their political orientation. The exploratory model indicated that political orientation influenced the way individuals responded to DTP framing. Specifically, framing only seemed to matter for individuals with a more liberal orientation and those who are moderate politically. These individuals were more likely to support DTP when it was defined as redirecting rather than eliminating. Liberal individuals are more likely to believe that DTP means reallocation (Jackson et al., 2023), therefore, when DTP was framed as redirect, these individuals are willing to support DTP, but when they are given a definition that goes against their belief (i.e., eliminating police), they are less likely to support DTP. Conservative support of DTP was lacking regardless of the frame, in line with previous research demonstrating low levels of support for DTP among conservatives (Jackson et al., 2023).

## 4 General discussion

The current research adds to the growing literature empirically examining public perception and support of DTP. While DTP has received increased public attention, vast protests, and increased calls for police reform across the country, empirical examination into public understanding and support for DTP is surprisingly lacking. Our results indicate an interesting narrative for the scientific literature, future scholarship, and public opinion and policy. In the absence of framing, our results replicate existing research examining DTP (Cobbina-Dungy et al., 2022; Fine and Torro, 2022; Jackson et al., 2023). Participants favor redirecting funds as the primary definition of DTP and are less supportive of the movement when they view it as eliminating funds (i.e., abolishing the police). Yet, in the presence of framing, an interesting trend emerges.

When framing DTP as redirecting funds, perceived support increases in comparison to the eliminating police frame. Notably, these framing findings hold even while controlling for individual differences such as familiarity with DTP, alignment of conceptualizations, and political orientation. However, our exploratory analysis examining the interaction between political orientation and framing suggests a more nuanced story. Framing only seems to matter for individuals that identify as extremely liberal or moderate. However, given liberal and moderate individuals make up a large portion of the United States. population combined (25 and 37%, respectively; Saad, 2022), the impact of such framing could have a substantial effect on policy changes.

It appears framing may not be as impactful for individuals identifying as extremely conservative. Given conservative and liberals have inherently different moral values (Kivikangas et al., 2021), perhaps a frame highlighting a value important to conservatives (e.g., saving taxpayer money by decreasing police budgets), would impact their support. The redirect theme in the current study highlights social services and community resources, an attribute that has been a historically liberal value (Sheldon and Nichols, 2009).

Taken together, these studies suggest there is much disagreement among individuals regarding DTP, but perhaps the disagreement stems from the divisive and ambiguous nature of the DTP slogan. After all, protesters themselves who are in support of DTP and police reform more generally have expressed concern with what the slogan implies (Cobbina-Dungy et al., 2022). Our results are some of the first to suggest the protesters’ concerns are not unfounded and that DTP implies different things to different people, and the resulting conceptualizations of these assumptions impacts both people’s support and understanding of DTP. It appears that if individuals are given a specific explanation of DTP (e.g., frame), emphasizing a relevant consideration related to what DTP would involve (e.g., redirecting police funds or eliminating police), public perceptions of the policy can be altered. This is particularly relevant for policymakers and social activists calling for change, who may want to first consider how their audience is conceptualizing DTP prior to attempting to garner support.

However, it is important to acknowledge that public support is not the sole factor that influences the success of public policy regarding police funding. For example, qualified immunity, a legal doctrine established in 1967, grants immunity to police officers accused of misconduct unless there is clear evidence that the conduct was unlawful *and* there is legal precedent for the misconduct (Novak, 2023; Qualified Immunity, n.d.). To further demonstrate the power of qualified immunity, the Supreme Court still upholds the doctrine despite public scrutiny, with 63% of Americans expressing support to abolish qualified immunity (Ekins, 2020). Another substantial consideration in the landscape of policing policy are police unions. Police unions can lobby for specific policies and make significant donations to politicians running for office (Datta, 2022), making it difficult, if not impossible, to create legislation at the highest levels. Thus, even if the public generally supports substantial changes to policing, there are significant legal and system barriers that contribute to how policing policy is both shaped and sustained across the country.

### 4.1 Limitations and future directions

Although this research revealed interesting findings and was built on a limited literature base, it is not without limitations. First, an online participant sample was used in both studies. Online samples are common in psychological research, and research shows that there is not much difference in data quality between MTurk and in-person samples (Kees et al., 2017). However, it is possible given the specialized and highly publicized topic of DTP, that our data missed an important segment of the population. Future researchers should work to replicate these findings with an in-person sample, perhaps allowing for more nuanced measures (e.g., behavioral observations or facial expressions). Secondly, the data was collected across a two-year period shortly following the death of George Floyd, a time when conversations around police reform, specifically DTP, was at its highest. It is possible and worth noting that the landscape for police reform may look quite different today. Future research on this topic should consider the impact of DTP frames now in 2024 and may want to consider longitudinal studies to examine the potential impact the period of heightened protests has on perceptions of DTP and framing.

Further, our research is limited by the lack of a control condition in Study 2 where an explanation of DTP is not provided to participants. Within such a condition, participants would indicate support for and provide definitions of DTP based on their own conceptualizations. Thus, the use of a control condition could have served as a baseline to investigate the extent to which the redirect or eliminate condition moved support up or down from baseline. With the current data, we are limited to the conclusion that participants in the redirect condition indicated greater support compared to those in the eliminate condition. Future research should explore this question to provide further evidence of the impact of the redirect and eliminate frames and the mechanism driving this effect. Lastly, the current study is limited in that it is unable to determine whether and to what extent the source of the frame may have an impact on perceptions of DTP. The source of the frames in the current research were neutral (i.e., there was not a source indicated). However, in real life, often, a source will be indicated, and individuals may use that source to obtain additional information that may impact their perception (e.g., credibility). Because police coverage is common in the media, it would be worth exploring the source of the frame in future research (e.g., *CNN* vs. *Fox News*). This comparison would also allow a more thorough examination of additional variables that may interact with political orientation and framing.

## 5 Conclusion

The current studies examined the public’s perception of DTP both with and without the impact of framing. Analyses indicated that in the absence of any frame, individuals tend to oppose DTP more than they support it (Study 1), and overall, individuals generally define DTP as decreasing funds to police departments (both with or without) a plan to redirect those funds to community resources, and/or as removing all funds from police (i.e., eliminating police). Although individuals clearly have differing views regarding DTP, the results of Study 2 suggest that individuals’ perceptions can change depending on how DTP is framed. More specifically, when DTP is framed as redirecting funds, individuals tend to be more supportive of DTP (compared to when it is framed as eliminating police), regardless of their political orientation, alignment with the emphasis frame, and familiarity with DTP. Emphasis framing of DTP also appears to not only impact support for the movement, but also how individuals conceptualize the slogan as whole. These results have important implications for the messaging surrounding police reform. Given these findings, the public may be more receptive to reform if the proposed changes involve redirecting funds to community resources. Police reform advocates should make their messaging clear, and if they choose to use the DTP slogan, an explicit explanation on their intended policy should supplement it.

## Data Availability

The raw data supporting the conclusions of this article will be made available by the authors, without undue reservation.

## Appendix

### Study 2 experimental manipulation operationalization

#### Eliminate condition

“Defund the police” means eliminating police. It means abolishing the police departments in our communities and doing away with law enforcement altogether. Supporters of “defund the police” want to completely disband, or eliminate, the police force and dissolve the local police unions. “Defund the police” means getting rid of our police force because there is minimal evidence that police surveillance results in reduced crime or prevents crime. By defunding the police – ending the institution of policing once and for all by eliminating traditional police forces – we will see decreases in crime and police violence.

In other words, “defund the police” represents an effort to end policing, in order to reduce unnecessary violent encounters between police and citizens. Supporters of “defund the police” believe that police reform is long overdue. Advocates believe that we have been given thousands of opportunities to make appropriate changes to police departments and have failed, and that we now know that more traditional policing is not the answer. Thus, supporters of “defund the police” believe that after failed attempts at reforming police practices, disbanding the entire police force is the best way to solve the problem.

#### Redirect condition

“Defund the police” means reallocating or redirecting funding away from the police department to other government agencies funded by the local municipality. Supporters of “defund the police” advocate for redirection of taxpayer money to social services that help fight the root causes of crime and poverty. “Defund the police” means shifting funding to social services that can improve things such as mental health, addiction, and homelessness. By defunding the police – reallocating funding away from police departments to other sectors of government, such as education – we will see decreases in crime and police violence.

In other words, “defund the police” means reallocating funding to trained mental health workers and social workers, in order to reduce unnecessary violent encounters between police and citizens. Supporters of “defund the police” believe that police reform is long overdue. Advocates believe that we have been given thousands of opportunities to make appropriate changes to police departments and have failed. Thus, supporters of “defund the police” believe that after failed attempts at reforming police practices, diverting funds toward underlying social issues is the best way to solve the problem.
